# Gynæcological Society of Chicago

**Published:** 1888-03

**Authors:** 


### SOCIETY REPORTS.

### TRANSACTIONS OF THE GYNÆCOLOGICAL SOCIETY OF CHICAGO.

### REGULAR MEETING, FRIDAY, NOVEMBER 18, 1887.

The President, HENRY T. BYFORD, M. D., in the Chair.

Professor W. W. JAGGARD exhibited

#### THE NEW NORMAL FORCEPS OF PROFESSOR LAZAREWITCH.

The instrument was presented to the Section on Obstetrics, Ninth International Medical Congress, and its full description can be found in the Transactions now in the printers' hands.

The forceps is straight, *i. e.*, without pelvic curve; the branches parallel; the lock, a tenon fitting into a mortise. Uncharitable critics might regard the instrument as an example of reversion to the original type of forceps. Johann Palfyn, a surgeon of Ghent, presented to the Academy of Paris, in 1723, a forceps, termed Manus Ferræ Palfynianæ, which, in many essential particulars, resembles the instrument devised by Lazarewitch after twenty years' experimentation.

The PRESIDENT presented the following specimens:

#### HYDRO-SALPINX.

The tube is dilated to a diameter of two and one-half inches. Before being put in alcohol the distended walls of the tube seemed as thin as tissue paper and the whole tumor almost transparent. The uterine end of the tube is pervious. The patient is 38 years old, and was never pregnant. She married fourteen years ago, was taken very sick in a short time, and did not live with her husband afterward. She has not been well since. She married again three years ago, lived with her husband eight months, and dates her severe illness from this marriage. She claims to have had one or two watery discharges from the vagina about the middle of each menstrual period, preceded by an aggravation of her symptoms and followed by relief. As her pains were on both sides, she concluded that the discharges came from both sides. At the first examination, made soon after a vaginal discharge, I found no tumor; at the second, I easily felt the dilated left tube. As you see, the ovaries are about twice their normal size. The right tube, which was small and imbedded in lymph on the posterior surface of the broad ligament, could not have been removed without tearing the ligament, and was not disturbed. The right ovary could not be pulled out at the incision, and was ligated with some difficulty. The recovery was easy and uninterrupted. The reason for the operation was that she was an invalid, unable to earn her living, and had no one to depend upon.

Dr. NELSON. Was there any suspicion that there had been any specific disease?

The PRESIDENT. From the history, I considered the disease to be of gonorrhœal origin.

### FIBRO-SARCOMA OF THE UTERUS AND BROAD LIGAMENT.

This specimen of fibro-sarcoma of the uterus is interesting as having been obtained by one of the most difficult hysterectomies on record. I have the following notes of the case:

Mrs. E. W., American, aged 40, widow, had been married twenty-two years, having borne four children, the oldest twenty years of age, the youngest twelve years old. Four times since the birth of her last child she has miscarried at the third month. Her present illness began six years ago, when she discovered an enlargement in the left inguinal region. There was no pain connected with it, but the last miscarriage, three years ago, was extremely painful. After recovery the pain in the left side subsided. One year ago, the growth began to increase in size, and has grown very rapidly since. There was dull, heavy pain through the pelvis. Two months previous to this time, an exploratory incision was made and a diagnosis given of fibroma of the uterus, with numerous pelvic adhesions. The surgeon did not think its removal practicable. On August 4, I removed the tumor. The operation was begun at 3.15 p. m. and completed at 7.15 p. m. The tumor, including the uterus, was about the size of a man's head. I found the whole anterior surface of the omentum firmly adherent to the abdominal wall above and the tumor beneath, making it almost impossible to get at the tumor, and certainly quite impossible then to make an ordinary exploratory incision. It required about three-quarters of an hour to get the omentum, whose veins were as large as goose-quills, ligated so that I could free it from the surface of the tumor. After enlarging the incision, I found a little space on the right where there were no adhesions. All over the left side the small intestines were adherent, lying flat on the surface, while above and on both sides the colon, throughout its whole length, lay as if plastered upon the tumor. Large blood-vessels could be seen running from the bowel onto the tumor. If I could have lifted the tumor sufficiently to put an elastic ligature about the uterus, I might have quickly enucleated it, but I could not stir it from its bed. It was a sarcoma that did not easily admit of enucleation, and bled profusely from the slightest wound. The patient was anæmic, had already lost some blood, and had been under ether for some time. But I had to go on, for I did not dare to close the incision with the already disintegrated surfaces free in the abdominal cavity. So by ligating dozens of places, cutting between some ligatures and enucleating under others, I finally got the tumor so that I could raise it a very little. Over two hours were thus consumed before I succeeded in freeing it above and tying the ovarian vessels of the left side. I then rapidly enucleated far enough down to apply an elastic ligature, using all of my hæmostatic forceps in stopping bleeding points. But the uterus had grown into the broad ligament and was firmly attached to the pelvis, so it could not be enucleated out of its vascular surroundings, but had to be ligatured at and against the pelvic brim on the left. The pedicle, as you see, was about the size of a man's thigh and is traversed on one side by the enlarged uterine cavity. It was treated by Hegar's extra-peritoneal method. The patient died forty-three hours after the operation, of exhaustion. The greater extent and vascularity of the adhesions, as compared with fibroma, were well illustrated here. The absence of menorrhagia in the history of the case corresponds with what is more often noticed in sarcoma.

Professor ETHERIDGE: Will you tell us how you freed the colon from the tumor, and whether the bladder was implicated?

The PRESIDENT: The bladder was not implicated. In freeing the colon, I took stitches through the capsule of the tumor and ligated separately the large vessels on the other side and cut between them. In some places I used hæmostatic forceps on the tumor side, in others I enucleated.

Dr. J. SUYDAM KNOX read the following report of three Pelvic Presentations, with Deep Laceration of the Perineum.

The three cases of breech delivery here reported occurred in close sequence in my private practice. Being unusually severe labors, and having certain points in common, they impressed me as being worthy of report. Taken collectively, they suggest discussion as to the management of labors with this presentation.

CASE I. Mrs. W., American, æt. 26, primipara, was taken with slight labor pains 4 a. m., May 7, 1887. At 2 a. m., May 8, Dr. Colton was called and found the breech presenting, S. L. A. As the os was but partially dilated, and the pains extremely irritating, he administered opium and chloral. Rest, with gradual dilatation of the os was thus obtained for the next twenty-four hours, when I first saw the case. Finding the membranes unruptured, and the os still not sufficiently dilated, the breech not being engaged, I advised non-interference and a continuance of the opium per rectum.

At the end of twenty-four hours I was again sent for. Water had been escaping for hours, the breech rested on the perineum, the patient was exhausted by seventy hours' labor, and the fœtal heart could not be heard. The doctor had made long and faithful efforts to assist the delivery, but the body was apparently impacted in the pelvis and would not advance. With great difficulty I succeeded in bringing down a foot, and at length delivered a lifeless twelve-pound boy.

The after-coming head caught in the superior strait and its extraction with forceps caused a laceration of the perineum to the right side of, and beyond, the anal sphincter. The soft parts were so swollen and contused that immediate stitching was deemed unadvisable.

The perineum was partially repaired by granulation during a tedious convalescence of five weeks. Secondary operation will be required.

CASE II. Mrs. M., a stout young Bohemian, æt. 22, fell in labor with her first child the morning of May 13, 1887, Dr. Michelet in charge. The first stage of labor was tedious and extremely painful. The membranes ruptured at the end of twenty hours. For three succeeding hours pains were severe and expulsive, when the patient became exhausted and labor ceased. Dr. Nelson was called in and advised stimulants, quinine, and opium. The patient slept several hours, awoke refreshed, and the pains returned with strength and regularity. Thirty-nine hours from the commencement of the labor, Dr. Nelson was again called in and I was requested to meet him. We found the breech resting on the perineum, the body firmly impacted in the pelvis, and the child dead. Persistent efforts at extraction had been made, leaving the vulva bruised and swollen, and the perineum rigid. The patient having been put under ether at the request of the others, I engaged a blunt hook in the anterior groin of the fœtus and gradually succeeded in extracting the body. The forceps were then applied to the after-coming head. Its delivery caused a laceration of the perineum through the sphincter, and about one inch up the recto-vaginal septum. The weight of the child was ten pounds.

Under the most disadvantageous circumstances, Dr. Nelson successfully closed the rent, using three silver sutures in the septum, and four in the perineum.

Antiseptic post-partum treatment was adopted, but the patient had septic fever. On the eighth day fæces escaped per vaginam. Examination revealed no union, and the stitches were removed. The patient was four weeks in bed. For two months she was confined to the house. She has but little control of the rectum to-day.

CASE III. Mrs. F., a robust American woman, æt. 36, was confined four years ago, in New York, with her first child. After seventy hours of distressing labor, a dead and mutilated child was instrumentally extracted. I could not learn whether craniotomy was practiced or not. On the morning of June 24, 1887, I was called to attend her in her second labor. I found the vertex presenting high up, L. O. A. The first stage of labor lasted thirty-six hours, when, the os being fully dilated, I ruptured the membranes. Hard expulsive pains succeeded for three hours, when finding the head did not engage, I attempted the high application of the forceps.

The blades were introduced and locked without difficulty, but each attempt at traction caused them to slip backwards over the crown of the child. Owing to the great obliquity of the head, this always occurred in spite of extreme depression of the handles and downward traction. Anæsthesia was then produced, the hand introduced, and podalic version easily accomplished.

The after-coming head became impacted in superior strait, requiring the forceps. Fifteen or twenty minutes passed before it could be disengaged and delivered. By depressing the handles of the forceps between pains, I succeeded in getting some air to the child, and thus finally delivered it alive. Weight of child, twelve pounds.

My best efforts could not save the perineum, which was torn through the anus and one inch up the recto-vaginal septum. After the delivery of the placenta, I repaired the laceration with two deep silver sutures. The upper stitch was inserted on a level with the lower vaginal commissure and passed entirely around the recto-vaginal rent. The second stitch closed the perineum above the sphincter ani. The rectal sphincter not having been repaired, the condition of the patient was similar to that after operation for anal fistula. The stitches were removed on eighth day, finding the perineum healed. The patient was up and about in two weeks, apparently as well as ever. October 13, the rectal tear not fully healed, but sphincter under full control. My impression is, that no further operation will be required.

In reviewing these cases, I was impressed with the danger to the fœtus in delay of delivery after the membranes have ruptured. Until then, breech presentations should not be interfered with, for the dilatation of the os is slowly accomplished, and the membranes should be kept intact as long as possible. This long first stage, however, is apt to exhaust the patient and make her irritable. We thus get spasmodic rigidity of the os tincæ and perineum.

After the rupture of the membranes and the protrusion of the breech into the vaginal canal, the rigid perineum is apt to cause the flexible body and folded limbs to pack in the pelvis, and delivery is retarded. If this retardation continues, tonic uterine contractions are induced and the child dies asphyxiated, or uterine inertia comes on and the fœtus slowly dies from compression. In the cases reported, the large size of the children, and the easy delivery of the after-coming heads, show that impaction was not due to disproportion between the pelvis and the fœtus.

These successive complications I believe are present in some degree in all breech presentations in primiparæ. The novelty of the situation, with its pain and anxious forbodings, tends to make such parturients nervous and irritable. Under ordinary conditions, breach presentations should be severely let alone until the breech has cleared the vulva. The bag of waters can scarcely be left too long unbroken; the folded body best prepares the way for the after-coming head, and, therefore, the extremities should not be brought down. Tractions lead to the extension of the chin and the passing of the arms above the head, and, therefore, ordinarily are unwise and vicious.

In cases, however, like the first two reported, there must be a departure from ordinary methods, to avoid the ill results that followed.

In a prolonged first stage, the resulting irritability of nerve and muscle can largely be avoided by a free use of opium guarded by belladonna. Rest is thus secured; strength conserved; the os tincæ becomes relaxed, and the perineum distensible.

After the rupture of the membranes, the descent of the breech should be encouraged by pressure upon the fundus uteri during each pain. At the same time, two fingers in the vagina depressing the perineum would prevent impaction, and increase the energy of the uterine contractions. Should impaction threaten, anæsthesia should be induced, the feet brought down, and tractions made; always, however, following the descending fœtus with compression of the fundus uteri. This outline of treatment is, of course, to be modified by the idiosyncrasies of the patient, or peculiarities of the case.

Each of the three patients reported was deeply lacerated. Each laceration was treated differently.

Case I was not operated upon. The soft parts were so ædematous and bruised as to forbid expectation of union. I believe this to have been an error of judgment. The œdema soon subsided, there was no sloughing, and partial repair, at least, with much comfort would have followed the closing of the wound. The mere apposition of the parts without union is a great advantage to the patient, as illustrated in Case II.

The latter was far less distressed during the first week of her lying-in, though her laceration was much more grave and no union resulted.

In Cases II and III, both lacerations passed through the sphincter ani, and about an inch up the recto-vaginal septum. In Case II, the whole wound was carefully closed with seven silver sutures. Non-union resulted. Septic fever was, however, present. In Case III, but two sutures were used, one closing the vaginal rent and the other the perineum. The deep anal fissure was left untouched. Good union followed.

I believe these opposite results had their origin in the management of the torn sphincter ani. In such deep lacerations this muscle is a born disunionist, and chafes under the restraint of a stitch. It should be excluded in the closing operation. The end sought is to restore the integrity of the perineum. Even the rent in the recto-vaginal septum, unless extensive, is of less importance.

Dr. D. T. NELSON: Perhaps it would be more appropriate for me to say something later in the discussion, but as I was cognizant of the first part of the history of the case, it may be interesting to give testimony as to the condition of the patient before Dr. Knox saw her and kindly assisted, for I assure you we wanted not only his brains but his muscles. The attending physician and myself had become completely exhausted in attempting to dilate the perineum and cervix and make ready for some one to deliver. I believe I never saw a more rigid perineum; the cervix had dilated fairly well, but slowly and tediously, assisted, I believe, by opiates—chloral had been given by the attending physician before I saw her. In theory and practice I have no doubt of the importance of aiding dilatation of the perineum when it does not dilate satisfactorily. This was one of those cases, rarely found, in which it was exceedingly difficult, almost impossible, to dilate. Considerable had been accomplished before Dr. Knox saw the patient, and yet I am sure he would be ready to testify that the perineum was not well dilated, and more, that it was not dilatable. It was ruptured before it was dilated. As to the wedging of the fœtus into the pelvis or into the lower strait, it was to me an interesting fact, and I think I have seen it several times before. It ordinarily means the death of the fœtus. Thus wedged in, when dead, the fœtus is, so to speak, a ball of putty which can be crowded by the forces above into a mass against the resisting medium, whatever it may be. Pains had been increased by the manipulations in dilating the perineum, and I think that is an important advantage in many cases of difficult labor, that by attempting to dilate the perineum the pains will be strengthened, just as they are ordinarily in a normal condition of things when the head, or whatever the presenting part is, reaches the perineum. You have the advantage of the reflex muscular contraction, which makes the pains very much more powerful. But we had become exhausted in attempts at dilatation before Dr. Knox saw the patient, so we got very little advantage in that way. What advantages have we gained by the experience? It seems to me the proper place for improvement in treatment of such a case is during pregnancy, or before pregnancy, even, and the question is whether or not we could do anything to make the muscular structure better in quality. It seemed to me one of those cases in which there was, to a large extent, absence of the development of muscular structures in the vagina and perineum which ordinarily follows pregnancy. We are all aware that pregnancy makes a great change in the vagina, vulva, uterus, and ovaries, and it seemed to me that change had not taken place as it should. There had not been the development of the muscular structures that would facilitate the delivery. She was much in the condition, when I first saw her, of a virgin. She was quite fleshy, and I think it is the experience of all of us that these are the patients whose perineums and other structures rupture; they do not dilate as well, perhaps from absence of muscular tissue, perhaps from the presence of fat. As to the restoration of the perineum, I remember well that we discussed the question whether it would be wise to attempt the immediate operation of the restoration of the perineum and other ruptured parts after delivery; my own thought, and I think it was the unanimous belief of those present, was that the woman would be no worse off if the immediate operation was performed, and probably would run less risk of sepsis, though we all doubted the satisfactory union of the parts. I heartily agree with Dr. Knox that, as a general rule, it is not wise for us to try to close the perineum and be thoughtless about the recto-vaginal septum and the sphincter ani. I should decidedly prefer, if I was to operate after another physician, to have the septum closed, and to have the opportunity of closing the sphincter and perineum. I think the first operation should be the closure of the septum. I think it will be found quite difficult to close the recto-vaginal septum, after the perineum and sphincter have been restored. I fully believe that the danger of sepsis and other serious complications are lessened by the immediate operation, even though the parts are so lacerated that we can hardly expect a satisfactory union, and I would do the primary operation unless the patient was so exhausted by the previous delivery as to forbid it.

Professor Chas. Warrington Earle: Such difficult breech cases as our attention has been called to this evening are not frequent in my practice. Indeed, when I have seen pelvic presentations terminated before I could reach the case, safely to both mother and child, it has sometimes occured to me that we exaggerated the dangers of these cases, and I have thought that the ordinary breech case gets along better without some doctors than with them. I think there is no doubt but that the temptation to extract quickly sometimes produces the complications we seek to avoid. The cases presented by Dr. Knox were indeed complicated, and, with his most able advisers, I believe the most judicious treatment was pursued, but I cannot see why episiotomy was not performed. This is an operation which, in my judgment, should not be done frequently or unadvisedly, but it occurs to me that here it would have been justifiable.

Dr. Nelson compares the condition of the child in the cavity of the pelvis to a mass of putty.

With such a condition of things as this—the child dead and the lower parts rigid—why waste time trying to deliver with forceps or anything else; why not perforate at once, reduce the size, and then deliver?

In regard to lacerations, I am an advocate of closing them all. I have never performed the immediate operation on the cervix, but I have witnessed it, and, if performed by a skillful operator, I think it justifiable. Certainly, it is our duty to do the immediate operation if the perineum is ruptured. I once saw Carl Braun's assistant do a craniotomy, in the course of which the cervix, a portion of the vagina, and the perineum were all torn. After the usual antiseptic precautions to the cavity of the uterus, he introduced retractors, and successively closed the rent in the cervix, then in the vagina, and then the perineum, using in all about sixty sutures.

I can easily see how in a small room, and with imperfect facilities, it would be impossible to do such an operation, yet the indications are to thoroughly and antiseptically and immediately close these lacerations.

Dr. D. T. Nelson: I would like to say a few words in reference to some of the points raised which Dr. Knox did not know. As to the use of hot vaginal douches, the hot sitz-bath was not used; it was practically impossible, on account of the absence of conveniences. A hot-water vaginal douche was directed and repeatedly used during the first stage of labor. A word further with regard to the septic conditions; I believe the woman had septic fever, but I believe that antiseptic precautions were fairly used, not as they might be in a hospital, or as they would be in our best private practice. Vaginal douches of an antiseptic type were repeatedly used during the first stage of labor, and after delivery iodoform was constantly thrown into the vagina and over the vulva. As to closure of the perineum by primary operation, and putting in too many stitches, I have had some experience in that direction, and fully believe that putting in a large number of stitches, and closing the parts perfectly, is a desirable thing to do, and I have repeatedly had it succeed most admirably. I never had as bad a result as this one, and the reason is partly explained by the fact that we were working at a decided disadvantage. We did not think it safe to put the patient on the table, where we could have had an opportunity to perform the operation satisfactorily. More than half the stitches were put in by feeling, and not by the eye, and you can judge that they would not be well put in, and accurate coaptation could not be secured. The expectation was not that there would be complete union, but that there would be less sepsis than otherwise, and I think the woman would have had far less chance of life if the operation had not been attempted. As to turning and delivering the child by his feet, I think any gentleman would have found it a difficult task to turn that babe and deliver it. As to dilating the parts before the instruments are put on, it seems easy, and in every other case of breech presentation I ever saw it was exceedingly easy to deliver the patient, but this was so extremely difficult I was very glad to have Dr. Knox present to assist, as well by his fingers as his valuable head. If I should ever meet another such case, I would be glad to have some gentleman of the society present, and if he can deliver with his fingers, they will be stronger than any that were present on this occasion; I feel sure there are no fingers in this society that could have delivered that breech.

The PRESIDENT: I would like to take exception to one of the main conclusions of the paper, that it is not well to try to do too much in closing the perineum. In those cases in which I have put in the most stitches the union has been the most complete. I recently put in twelve stitches and got complete union in a laceration extending into, but not through, the sphincter ani. In another, extending through the sphincter ani, I obtained complete union by using fifteen stitches. I preserved a piece of the perineal centre, which was hanging out like the end of a finger, by stitching it back with buried juniper catgut. The reasons for failure are that the operation is poorly done, or imperfectly cared for afterwards. If the operator uses carbolized catgut, he will be sure to have occasional failures. I use buried sutures of juniper catgut in the recto-vaginal septum, instead of rectal sutures, and then stitch the vaginal edges, exactly as they belong, with the same material. The lowest external stitch should be taken low down and through the ends of the torn sphincter, as recommended by Emmet. Either this or the next one should pass deep enough into the perineal body and near enough to the rectal mucous membrane to sustain the rectal pressure or traction as far as possible. If these stitches be of silk-worm gut or silver, they will not give way, when properly and aseptically taken. I think the mistake is sometimes made of cutting off too many irregularities, instead of fitting them together. After the parts are united, we should, if the rectum has been lacerated, bind up the bowels for several days, and at first use plain vaginal douches, not carbolized ones, from three to five times in twenty-four hours. Beginning with the third and fourth day, carbolized douches should be employed. If we take all this care, the same as for the secondary operation, we will, unless the parts have been too badly bruised, have the same success.

Dr. J. S. KNOX: In regard to the infection of these patients, Dr. Cotton told me his patient had no septic fever or rise of temperature, and he thought convalescence was delayed on account of the laceration of the parts. Dr. Michelet's patient had septic fever. The stitches were removed on the eighth day, and union was found not to have taken place. In my own case, the woman made a prompt recovery. I kept her in bed two weeks, because I had stitched the perineum; there was no rise of temperature, no signs of sepsis; she had a normal lying-in. I introduced my hand into the uterus and turned the child. I used no intra-uterine douche, but used an antiseptic vaginal douche, two or three times a day. In reply to Dr. Earle—the physicians were not trying to save the perineum, as the lacerations were not at all expected. I have repeatedly applied the forceps to the after-coming head in breech presentations, and always expect to deliver the head without laceration of the perineum. It has been my experience that when a delivery of the after-coming head is attempted instrumentally, it comes suddenly and unexpectedly; you make strong traction, and the head flies upon the perineum and out in the world in a moment, and I think the lacerations of the perineum occurred in this sudden popping-out, so to speak, of the after-coming head. Dr. Byford speaks of keeping the bowels bound up after operations on the perineum. I met with greatest success in keeping the bowels open; my inflexible rule is, after stitching the perineum, to administer to my patients, from the start, Friedrichshall or Hunyadi water. I like them to have a stool once in twenty-four hours without the use of an enæma; I also like my patients to pass urine naturally, and then, as soon as the patient has urinated, to douch out the vagina and the wound. I think it is not judicious to keep the bowels constipated. In regard to Dr. Nelson's criticism of my conclusion about too much being attempted, I spoke only of this one case. I believe that all lacerations should be closed, if possible, and that every care should be taken to perform an immediate and complete operation. When, however, the conditions are such that one cannot make a complete operation, I should much prefer to close the perineum, leaving the sphincter-ani muscle unclosed, to even leave the septum not stitched, rather than the perineum, because I believe that the results of a laceration of the perineum, unrepaired at the time and left for a secondary operation, are quite serious. There seems to be a readjustment of all the organs of the pelvis; the position of the bladder, rectum, and uterus are not the same as before, and the longer the secondary operation is delayed the greater are the displacements. In this operation, I said too much was attempted, because an appropriate and satisfactory operation could not be done under the circumstances.

All the surroundings were such that all operations were done with a great deal of difficulty; in fact, Dr. Nelson made half his stitches by touch, and not by sight. In my own case, I was alone, with no assistant but an ignorant Irish nurse, and with a hysterical patient. In the case operated upon by Dr. Nelson, I think, if he had used less skill, he would have had a good perineum. Speaking from my personal experience, I think that manual dilatation of the os in labor is injurious; I believe most thoroughly in the septic inoculation of the uterus by attempts to dilate the os in labor, even before the membranes are ruptured. When the os has become rigid, I occasionally introduce two fingers and try to retain what dilatation has been accomplished by pains; but the dilatation of the os, I think, is a vicious practice in ninety-nine cases out of one hundred, but by dilating the perineum when it is rigid I have saved a rupture, in breech as well as vertex presentations. If impaction of the fœtus and laceration is accomplished largely by rigid perineums, I think it is proper for the physician to do this operation, and I know that it does bring on more regular pains.

Professor Earle asked if these women were subjected to a long hot sitz-bath as a means of assisting dilatation.

Dr. Knox: They were not. There are many things that have suggested themselves to me in connection with these cases. I believe that if hot vaginal douches, hot sitz-baths, and hot applications over the abdomen had been used, they would have been more satisfactory.

Dr. Bayard Holmes read a paper entitled, "A Primary Myoma of the Broad Ligament and a Table of Seventeen Collected Cases," which appeared in the February number of this journal.

